# Influence of Dose Intensity in Consolidation with HIDAC and Other Clinical and Biological Parameters in the Survival of AML

**DOI:** 10.1155/2020/8021095

**Published:** 2020-06-24

**Authors:** Ricardo Ballesteros-Ramírez, Sandra Quijano, Julio Solano, Camila Ordoñez-Reyes, María V. Herrera, Raúl Murillo, Susana Fiorentino, Mónica Arevalo-Zambrano

**Affiliations:** ^1^Group of Immunobiology and Cell Biology, Faculty of Sciences, Pontificia Universidad Javeriana, Bogotá, Colombia; ^2^Hematology Service, Hospital Universitario San Ignacio, Bogotá, Colombia

## Abstract

**Background:**

The impact of the dose intensity administered in consolidation in Latin America is unknown. This study aimed to evaluate the relative dose intensity (RDI) in consolidation and its impact in overall survival.

**Methods:**

A retrospective study of 86 patients with AML who were diagnosed between 2010 and 2016 with a 2-year follow-up in a fourth-level Colombian hospital was carried out. Clinical characteristics were reported, Kaplan-Meier was used for estimating the overall survival, and Cox regression was used for multivariate analysis.

**Results:**

The median overall survival (OS) was 20.83 months, and the median event-free survival (EFS) was 16.83 months. 64.3% of the patients achieved remission after the 7 + 3 chemotherapy induction treatment. Patients under 30 years of age, with white blood cell counts less than 100.000 cells/mm3 who responded to induction treatment had a better OS. Additionally, patients receiving an RDI greater than 0.75 of the planned consolidation dose had better survival. The prognostic variables with impact in the OS were the leukocyte count in peripheral blood at diagnosis, the RDI in consolidation treatment with HIDAC and the response obtained after induction.

**Conclusion:**

This retrospective study allowed us to know the epidemiology of AML in a reference Colombian Hospital. Additionally, in our knowledge, it is the first study that reports the RDI in consolidation with HIDAC in Latin America as a prognostic factor that directly impacts the OS.

## 1. Introduction

Acute myeloid leukemia (AML) is defined by the malignant clonal expansion of progenitor cells coupled with a differentiation arrest [1, 2] Currently, the identification of certain molecular alterations such as mutations, together with the immunophenotype information at diagnosis and during the posttreatment clinical follow-up, has allowed a risk redefinition of patients with AML, approximately 55% of patients with AML show chromosomal genetic alterations [1–3].

According to the Colombian National Cancer Institute (INC) for the 2007-2011 period, 1,256 new cases of acute leukemia were reported with an incidence of 6.0 cases per 100,000 population, with a 5-year survival of 23.6% [4]. In 2018, the program for global surveillance of cancer survival (CONCORD), published the statistics in which Colombia was included with records from four cities, reporting survival rates at 5 years for AML of 31.8% [5]

Cancer outcomes are highly influenced by inadequate funding; inequitable distribution of resources and services; inadequate numbers, training, and distribution of health-care personnel and equipment; lack of adequate care for many populations based on socioeconomic, geographic, and ethnic factors; fragmentation in health services, administrative barriers, availability of access to the service, and type of coverage in the health plan are some but not all the challenges caring for patients with cancer in Latin America and the Caribbean [6, 7]. It is well known that delays in initiating treatment and abandonment of therapy impact the OS. In pediatric acute leukemia, a study showed that patients who received their therapy without excessive delays had similar results to those achieved in the countries of reference for this disease [8].

The clinical outcomes of AML are influenced by risk classification, response to treatment, and by the intensity of chemotherapy doses received particularly in consolidation. In AML, the treatment includes an induction protocol with an anthracycline for 3 days plus 7 days of ARA-C (7+3). This is followed by 4 consolidation protocols with high doses (3 g/m2) of ARA-C (HIDAC) for 3 days. The patient must attend at the appropriate times to receive the protocol to achieve the best outcomes, but delays in the administration of treatments in Latin America are known. These delays impact the relative dose intensity (RDI), which represents the ratio of the amount of drug administered compared to the planned dose within a set period of time [9]. The RDI is considered as a measure of treatment quality, and a strong correlation with clinical outcomes has been seen. Currently, there is little information in the literature regarding the evaluation of this metric and its impact. The objective of this study was to characterize AML in a hospital in Colombia, considering different clinical and biological variables used in risk stratification, and to relate the RDI in consolidation treatment with OS.

## 2. Methodology

A retrospective analysis of the adult patients who were diagnosed and treated with AML in the period from 2010 to 2016 was performed. The patients were identified using the electronic health records from the Hospital Universitario San Ignacio (HUSI) in Bogota, Colombia. The following inclusion criteria were used for the patient selection: patients who were diagnosed by the institution's adult hematology service, patients who received induction treatment, and patients with clinical data available in the clinical history. Patients with acute promyelocytic leukemia (APL) were excluded from the analysis.

The guidelines for the classification of hematopoietic tumors defined by the WHO (2016) were used, and the induction and consolidation of the chemotherapy protocol received by the patients was reported. The dosage received and the time interval between each cycle (induction and consolidation) was also considered for calculation of the RDI.

The response criteria were defined considering the recommendations of the European Leukemia Net (ELN) 2017. Patients with treatment failure were classified into the following groups: primary refractory disease, death in aplasia, and death of undetermined cause. The risk category was defined according to the types of genetic abnormalities present in each leukemia, and in this way, the patients were grouped into the favorable, intermediate, and adverse risk categories, considering the criteria described by the ELN 2017 [2].

OS was defined as the time the patients diagnosed with the disease were still alive following diagnoses. EFS was defined as the period that elapses after finishing a treatment, during which the patient survives without signs or symptoms of the disease. The event was defined as relapse or death at the follow-up time.

This study was approved by the ethics committee of the Hospital Universitario San Ignacio in Bogota, Colombia (FM-CIE-0206-16).

### 2.1. Statistical Analysis

The statistical package SPSS, version 23 was used for data analysis. A descriptive analysis of the frequencies was performed for the qualitative variables, and the quantitative variables were expressed as the median and the interquartile range with a 95.0% confidence interval. The method of Pardo et al. [10] was used to adjust the value of those patients in whom there was a loss in the follow-up. For 6 patients in whom it was not possible to find the death date exactly, half of the year was imputed as the date of death, the value of OS found with this date was adjusted with the median survival values found for patients who died in HUSI.

For the response to treatment, the percentage of patients who achieved complete remission after the induction protocol with the different treatment protocols used was considered.

The relative dose intensity (RDI) was calculated as reported by Yamaguchi et al. (2011) where the dose received by the patient is related to the standard dose of the protocol that was adjusted for the number of weeks. 0.75 was considered as a cut-off point for the analysis. For the calculation of the OS and EFS time, a closed cohort was used with a 2-year follow-up using the Kaplan-Meier method. These values were reported as the median survival in months and standard errors with 95% confidence intervals. The survival graphs were compared using the log-rank test for the different stratification used.

Cox regression was used for multivariate analysis. It included those variables that had some differences or presented clinical significance in OS.

## 3. Results

### 3.1. Patients Clinical Characteristics and Response to Treatment

86 patients treated at the Hospital Universitario San Ignacio with newly diagnosed AML were identified in a period of 7 years (2010-2016). The ratio of men and women was of 1 : 1.23 (55.8% vs. 44.2%); the median age at diagnosis was 52 years (95% CI: 17-82 years), where it is highlighted that 37.2% of the patients were older than 60 years. Within the hematological and cytogenetic parameters, 8.2% of the patients presented hyperleukocytosis with leukocyte counts greater than 100,000 cells/mm3 and abnormal karyotype in 25 patients (29.0%). Considering the genetic abnormality identified in the tumor cells in the bone marrow sample at the time of diagnosis, 7% of the patients were grouped at favorable risk, 50.0% at intermediate risk, 22.1% in adverse risk, and 20.9% could not be classified. According to the morphological (myelogram) and immunophenotypic criteria by multiparameter flow cytometry, the most frequent AML was acute monoblastic leukemia (24.4%), followed by AML with and without maturation (15.1%). Additionally, AML secondary to MDS accounted for 12% of cases and postchemotherapy AML corresponded to 3.5% (Table 1).

Stratification of patients according to risk showed that 22.1% of patients presented genetic alterations associated with poor prognosis, and 50.0% of patients were classified with intermediate-risk and 7.0% with favorable risk. 20.9% did not present associated genetic alteration and/or the karyotype was not determined.

Regarding the treatment administered, 68 (79.1%) of the 86 patients included in the study received treatment with the 7 + 3 protocol. Of this group, 67% achieved remission after the induction protocol. On the other hand, 16 patients who did not reach remission with the 7 + 3 protocol, underwent reinduction with high doses of cytarabine (HIDAC), reaching a response in 81.3% of them. 2 patients (2.3%) received AIDA-PETHEMA due to an initial diagnosis of acute promyelocytic leukemia that was subsequently ruled out, and 7 patients received support therapy (8.1%) (Table 2). Of the group of patients who received treatment with curative intent, 10 patients (14.7%) had a minimal negative residual disease, and 65 patients (85.3%) had a positive residual minimum disease measured by flow cytometry. When comparing the median survival between these groups, no statistically significant differences were found.

Of the total patients, 49 (57.65%) received consolidation treatment with HIDAC, 13 patients received 1 cycle (26.5%), 6 patients 2 cycles (12.2%), 14 patients 3 cycles (28.6%), and 16 patients 4 cycles (32.7%). Because the bone marrow transplant is performed in several institutions, which implies the loss of follow-up in some cases, only 12 transplants were registered, of which 5 were performed institutionally and 7 were extrainstitutional.

### 3.2. Overall Survival

**Figure 1:**
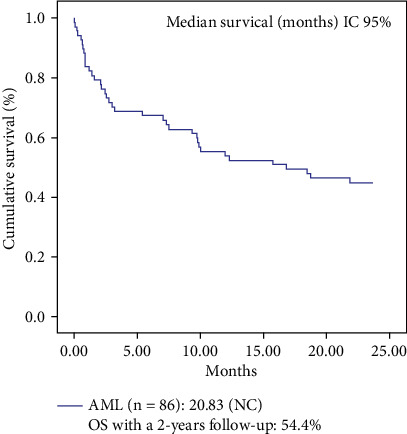
Overall survival for cohort of AML patients.

A closed cohort (2010-2016) was used to calculate the overall survival (OS) with a 2-year follow-up (Figure 1). The median was 20.83 months (54.4%). The OS with open cohort and without adjusting by the method of Pardo et al. can be observed in Figures S1–S2 (see Figures S1–S2 in the Supplementary Material).

**Figure 2:**
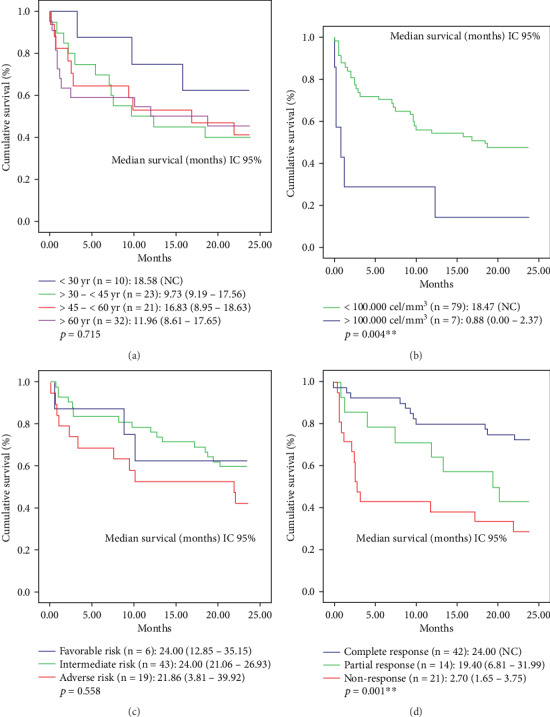
Overall survival for AML patients stratified by (a) age groups, (b) leukocyte count, (c) cytogenetic risk, and (d) response groups after induction.

The analysis carried out by age groups in the stratification (Figure 2(a)), the patients under 30 years had a better OS when compared with the other groups. It is noted that at 2 years, the group of patients aged between 30 and 45 years, had a median survival lower than the other categories. For other clinical variables, it is important to highlight that the median OS was better in groups with counter of WBC below 100,000 cells/mm3 (OS of 18.47 months) vs. patients with counts greater than 100,000 cells/mm3 (OS of 0.88 months) (Figure 2(b)).

The analysis of the OS by cytogenetic risk shows that the groups of patients with favorable and intermediate-risk have a similar OS. When compared with the high-risk group, a difference in OS of approximately 3 months is observed (Figure 2(c)). Finally, when the responses after induction were compared, it was found that the group that showed a complete response had a higher OS than the nonresponse group which had an OS of only 2.70 months (*p* = 0.001) (Figure 2(d)).

Another prognostic impact variable analyzed was the 2-year event-free survival (EFS), which was 16.83 months. When this variable was calculated considering the age groups, a behavior similar to that described in the OS is observed, where patients under 30 years of age show an EFS greater than the other groups (median not calculable). Similarly, the EFS calculated based on the leukocyte counts obtained in the blood count shows that the group with hyperleukocytosis had a lower EFS with a value of 0.833 months when compared to the 8.73 months found in patients with counts less than 100,000 cells/mm3 (*p* = 0.003) (data not shown). The EFS evaluated through risk and response does not show significant differences between the different categories.

### 3.3. Relative Dose Intensity (RDI)

To calculate the influence of the relative dose intensity (RDI) in consolidation with the HIDAC protocol, a cut-off point for the RDI of 0.75 was used ensuring that there were no significant differences in other prognostic clinical characteristics between the two groups that could influence the survival outcome.

The OS was calculated with a 2-year follow-up.  Table 3 shows the results of OS according to RDI. An RDI of 0.75 was observed, and the difference for 2-year OS was of 14.67 months between the two groups analyzed.

### 3.4. Multivariate Analysis

The multivariate analysis included the clinical variables that presented significant differences in the OS. The variables included were prognostic groups defined according to the genetic alterations detected at the time of diagnosis, leukocyte counts in the peripheral blood sample at diagnosis, consolidation dose intensity, and response groups. The analyzed variables, total white blood count, dose intensity in the consolidation treatment, and response groups were factors that together impact the overall survival, as can be observed in Table 4.

## 4. Discussion

Colombia is classified as an upper-middle-income country; however, income inequality is high, with 46% of the population living below the national poverty line ($4 US dollars/day) and 16% of the population unable to satisfy basic nutritional needs in 2009 [8]. The HUSI is a private hospital in Bogota, Colombia, that serves as a referral center for patients with known or suspected cancers of all types. As a fourth level referral center, the HUSI attends patients from different economic and social backgrounds as well from other cities all the country. In the patient cohort evaluated, a 2-year OS of 54.4% was found. This value is higher than reported by other studies in Colombia where an approximate OS of 28.0% is described [11, 12]; the different OS could be explained by the improvement of treatments and care in AML through the time. The 2-year EFS was 16.83 months, which is similar to other countries with social conditions similar to those of Colombia [12, 13].

In Latin America, survival has been always lower than in high-income countries. Brazil has reported different OS for AML; the 5-year OS has been between 17.0% and 23.0% [14–16]. In Mexico, a 25 months OS was described [17], and in a single institution study in Chile, the median OS was 24 months [18]. Despite, the follow-up was 2 years in our research, the nature of the outcomes tends to be similar. One reason is the different economic status of the patients who attend the treatment in the hospital; it is well known that low socioeconomic status, including poor housing conditions, low per capita income, and energy consumption, was associated with a higher risk of relapse in acute leukemia [15]; even though the socioeconomic status was not measured in our study, the patients have a heterogeneous background and depend on the coverage healthcare can carry out to abandonment and delays in the therapy which are related with poor outcomes [8].

A gender-based distribution of the data was also observed, where the men were diagnosed more than women with a ratio of 1 : 1.23, a finding that is consistent with that reported in international studies [19, 20] but no with Latin American and Colombia studies [11, 12, 15, 16, 21]. The problem with this incidence and the results obtained is that our study is not based in a demographic registry and the sample size is limited, which cannot be reflecting the real behavior in our population.

In our cohort, 37.2% of patients were over 60 years old. This result is lower than what is reported in studies in countries such as the United States and the United Kingdom where figures of 70% are reached (“Acute myeloid leukemia (AML) statistics |Cancer Research UK”; Howlader et al., 2017). In Colombia and Latin America, the age distribution is heterogenous; in some studies, the median age and the patients over 60 years are similar to our study [14, 18, 22], but in other reports, the patients described are younger than expected, where the median age was around 40 years [11, 12, 15–17, 23], which could be associated with the design of the studies and particularities in the Latin America population in the presentation of the disease and that the majority of the studies are not demographic-based.

An abnormal karyotype was found in 29.1% of the cases, a value that is below international reports, where around 55.0% of patients with AML have an abnormal karyotype [24]. However, it is similar to national registries where the abnormal karyotype is around 25.0-35.0% [11, 12]. It is important to know that in about 22% of patients no metaphase growth was obtained due to technical problems in the analyzes or the karyotype study was not requested by the physicians. When comparing OS in cases with favorable risk versus cases with unfavorable risk, no significant differences were found that may be related to the sample size and the follow-up time of the patients that was only 24 months. Another explanation may be due to the exclusion of patients with APL, which are analyzed in other studies and may increase OS times [11, 12, 25, 26] and that the sample size was limited in our study, then the risk groups were not enough to detect the different OS.

The chemotherapeutic protocol used was mainly with anthracyclines and ara-C in the 7 + 3 protocol, where 67.0% of the patients achieved postinduction remission. This response rate is similar to what is obtained at the national and international levels, where it is between 65.1% [11] and 70.0% [27], respectively. Brazil studies with the same chemotherapeutic protocol have reported a postinduction hematological remission of 57.0% [14], 40.0% [15], and 43.0% [16]; Mexico described a 84.0% remission in a 21 patients study and Chile between 65.0 and 81.0% [18].

The consolidation therapy that was used was HIDAC and on average 3 consolidation cycles were administered after induction. However, only 49 patients of the initial 86 who received induction therapy were consolidated with chemotherapy. This fact may be associated with changes in the health service provider that leads to patients being treated in other institutions or to the health system that delays the times to provide the chemotherapy. For that, we decided to explore the RDI as a factor that could influence the OS. In AML, it is not a specific cut-off point for the RDI in consolidation. Therefore, three different cut-off points (0.90, 0.85, and 0.75) were made, based on what is reported in other hematological malignancies [9, 28, 29]. It was found that for the RDI points of 0.90 and 0.85, the groups formed had significant differences in prognostic factors. Nevertheless, for an RDI of 0.75, these prognostic factors were balanced. The group of patients who had an RDI greater than 0.75 in consolidation therapy had a 2-year OS higher in 14.67, a finding that has not been reported until now. This is consistent with international studies that, although they do not evaluate RDI, do show that a lower dose of ara-C in consolidation affects disease-free time [30]. Although there are few studies of the RDI in consolidation with HIDAC, Fagundes et al. showed that patients who reached CR and received one course of HIDAC for postremission therapy had similar probabilities of OS and EFS to patients treated in developed nations and this could depend on the socio-economical status of the patients, situations that we did not evaluate in our study but has to be considered for future studies.

## 5. Conclusions

This study shows the clinical characteristics of patients with AML in a Colombian institution with a 2-year follow-up. The intensity of consolidation doses is reported for the first time as a prognostic factor, a situation that must be evaluated in subsequent studies for decision-making. It should be noted that these results must be interpreted carefully, since there may be clinical and nonclinical situations that are affecting the RDI, such as the case of therapeutic adherence impacted by decisions of the insurer, by the patient himself or by dose adjustments by clinical criteria. These conclusions must be taken with cautions based on the limited sample size.

## Figures and Tables

**Table 1:** Description of the clinical characteristics of patients with AML.

Characteristic	*n* (%)
Sex (men)	48 (55.8)
Age (years), median (interval)	52 (17-82)
<30 years	10 (11.6)
30–45 years	23 (26.7)
46–60 years	21 (24.4)
>60 years	32 (37.2)
Immunophenotype	
Undifferentiated acute myeloblastic (M0)	8 (9.3)
AML with minimal maturation (M1)	13 (15.1)
AML with maturation (M2)	13 (15.1)
Acute myelomonocytic leukemia (M4)	4 (4.7)
Acute monocytic leukemia (M5)	21 (24.4)
AML, not specified (NOS)	14 (16.3)
AML and MDS, therapy related	
AML with characteristics of MDS	10 (12.0)
AML secondary to chemotherapy	3 (3.5)
Karyotype	
Normal	42 (48.8)
Abnormal	25 (29.1)
No growth	7 (8.1)
Not requested	12 (14.0)
Cytogenetic/molecular alterations	28 (32.6)
FLT3 ITD	4 (14.2)
NMP1	1 (3.6)
FLT3 ITD with NMP1	3 (10.7)
t(9;22)(q34.1;q11.2)	1 (3.6)
Del 5q	3 (10.7)
46 XX -7 +7q- (Del7q)	1 (3.6)
46 XY, right (16)(17)	1 (3.6)
46 XY, -10, +10	1 (3.6)
45 XY, t(14p17p), -14,-17	1 (3.6)
Trisomy 8	4 (14.2)
Trisomy 12	1 (3.6)
+6 +8 +12	1 (3.6)
46, XY/49, XY, -1, -4, -5, -11, -12, 17, +mar,+mar,+mar,+mar.+mar,+mar,+mar,+mar,+mar	1 (3.6)
Inv (16)	1 (3.6)
t (8;21) (q22;q22.1)	4 (14.2)
Cytogenetic risk	
Adverse	19 (22.1)
Intermediate	43 (50.0)
Favorable	6 (7.0)
Undetermined	18 (20.9)
WBC count (mm3), median (interval)	35090 (500-298400)
Hemoglobin (g/dl), median (interval)	8.85 (3.2-16.06)
Platelet count (mm3), median (interval)	79460 (2800-570000)

**Table 2:** Treatment characteristics in the cohort of AML patients.

Treatment	*n* (%)
High intensity treatment	
Induction 7 × 3	68 (79.1)
AIDA PETHEMA	2 (2.3)
HIDAC	1 (1.1)
Low-intensity treatment	
5 azacitidine	4 (4.7)
Others	4 (4.7)
Support treatment	7 (8.1)

**Table 3:** OS according to the RDI received in consolidation.

RDI = 0.75	Median survival with a 2-years follow-up
<0.75 (*n* = 26)	9.33 (0.00–24.15)
>0.75 (*n* = 23)	24.00 (15.82–32.18)
*p*	0.266

**Table 4:** Multivariate analysis to assess risk factors that affect the OS.

Variable	HR	IC 95.0%	*p*
WBC counts in PB	1.008132	1.001553-1.014755	0.015∗
RDI	1.4908643	1.3395053-1.709703	0.000∗∗
Complete response	1.35011	1.097194-1.661326	0.005∗∗

## Data Availability

The clinical data used to support the findings of this study are restricted by the Institutional Review Board from Hospital Universitario San Ignacio in order to protect patient privacy. Data are available from Dr. Margarita Manrique, mmmanrique@husi.org.co, for researchers who meet the criteria for access to confidential data.

## Supplementary Materials

Supplementary Materials Figure S1: overall survival for AML calculated with the open cohort. Figure S2: overall survival for AML calculated with the date and vital state of the last record in the medical record.
