# Supplementary material for: Influence of Dose Intensity in Consolidation with HIDAC and Other Clinical and Biological Parameters in the Survival of AML
Source: J Cancer Epidemiol. 2020 Jun 24;2020:8021095. doi: 10.1155/2020/8021095 (PMC7333041; doi:10.1155/2020/8021095)
Supplement: Supplementary Materials — Figure S1: overall survival for AML calculated with the open cohort. Figure S2: overall survival for AML calculated with the date and vital state of the last record in the medical record. [file 8021095.f1.pptx]

## Slide 1
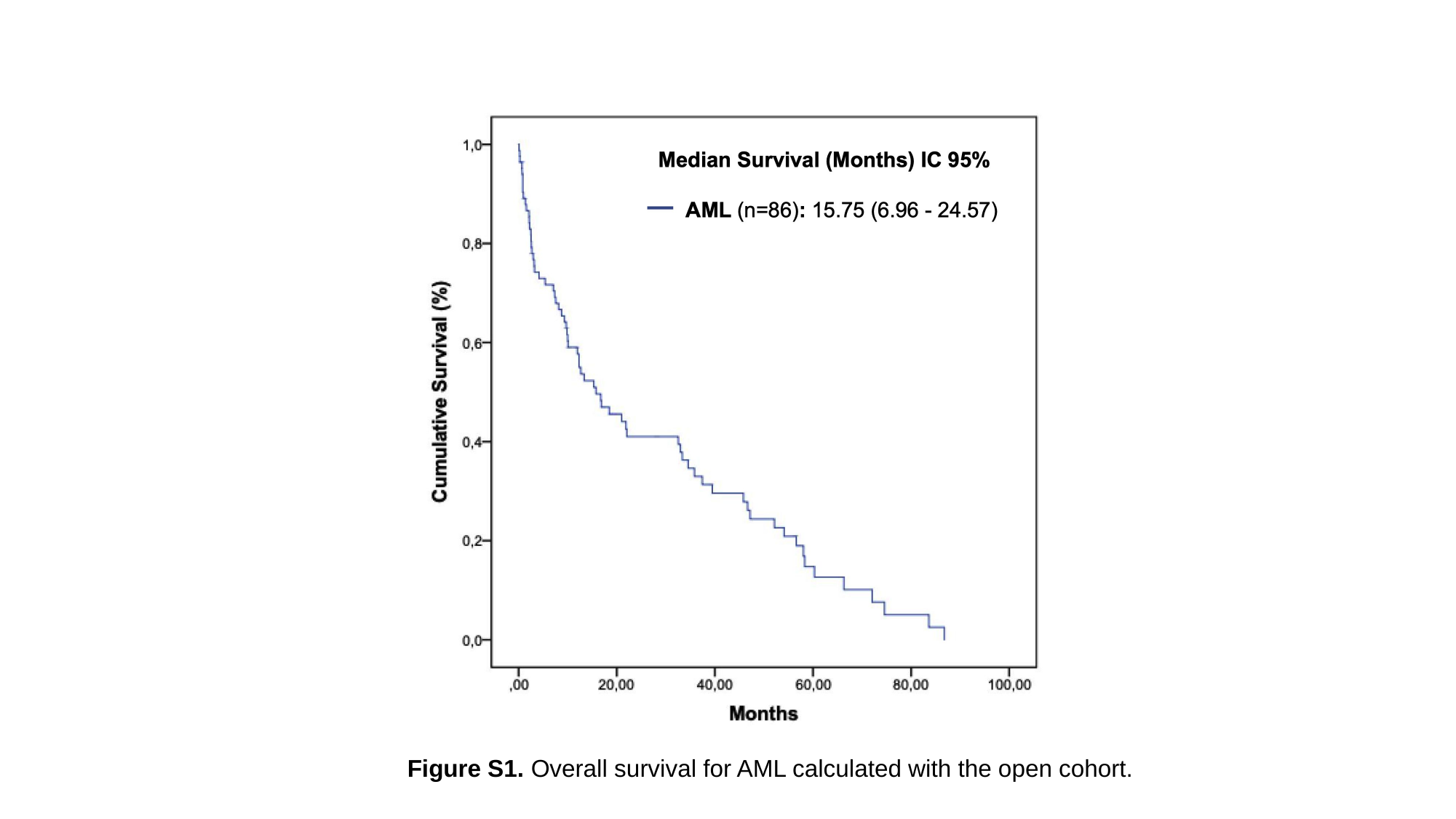

Figure S1. Overall survival for AML calculated with the open cohort.

## Slide 2
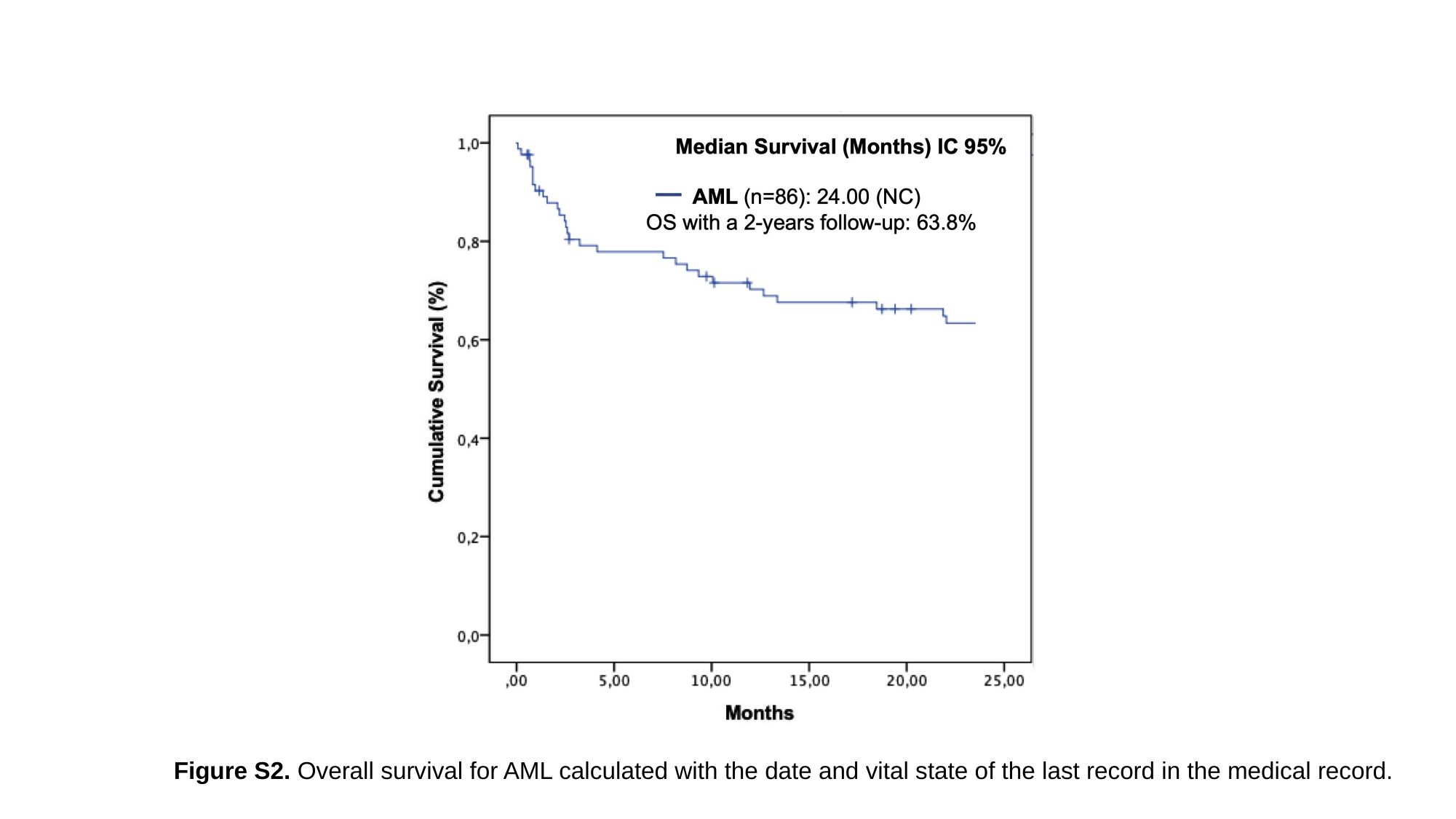

Figure S2. Overall survival for AML calculated with the date and vital state of the last record in the medical record.
